# RHOA Is a Modulator of the Cholesterol-Lowering Effects of Statin

**DOI:** 10.1371/journal.pgen.1003058

**Published:** 2012-11-15

**Authors:** Marisa W. Medina, Elizabeth Theusch, Devesh Naidoo, Frederick Bauzon, Kristen Stevens, Lara M. Mangravite, Yu-Lin Kuang, Ronald M. Krauss

**Affiliations:** 1 Children's Hospital Oakland Research Institute, Oakland, California, United States of America; 2 Sage Bionetworks, Seattle, Washington, United States of America; Vanderbilt University, United States of America

## Abstract

Although statin drugs are generally efficacious for lowering plasma LDL-cholesterol levels, there is considerable variability in response. To identify candidate genes that may contribute to this variation, we used an unbiased genome-wide filter approach that was applied to 10,149 genes expressed in immortalized lymphoblastoid cell lines (LCLs) derived from 480 participants of the Cholesterol and Pharmacogenomics (CAP) clinical trial of simvastatin. The criteria for identification of candidates included genes whose statin-induced changes in expression were correlated with change in expression of *HMGCR*, a key regulator of cellular cholesterol metabolism and the target of statin inhibition. This analysis yielded 45 genes, from which *RHOA* was selected for follow-up because it has been found to participate in mediating the pleiotropic but not the lipid-lowering effects of statin treatment. *RHOA* knock-down in hepatoma cell lines reduced *HMGCR, LDLR*, and *SREBF2* mRNA expression and increased intracellular cholesterol ester content as well as apolipoprotein B (APOB) concentrations in the conditioned media. Furthermore, inter-individual variation in statin-induced *RHOA* mRNA expression measured *in vitro* in CAP LCLs was correlated with the changes in plasma total cholesterol, LDL-cholesterol, and APOB induced by simvastatin treatment (40 mg/d for 6 wk) of the individuals from whom these cell lines were derived. Moreover, the minor allele of rs11716445, a SNP located in a novel cryptic *RHOA* exon, dramatically increased inclusion of the exon in *RHOA* transcripts during splicing and was associated with a smaller LDL-cholesterol reduction in response to statin treatment in 1,886 participants from the CAP and Pravastatin Inflamation and CRP Evaluation (PRINCE; pravastatin 40 mg/d) statin clinical trials. Thus, an unbiased filter approach based on transcriptome-wide profiling identified *RHOA* as a gene contributing to variation in LDL-cholesterol response to statin, illustrating the power of this approach for identifying candidate genes involved in drug response phenotypes.

## Author Summary

Statins, or HMG CoA reductase inhibitors, are widely used to lower plasma LDL-cholesterol levels as a means of reducing risk for cardiovascular disease. We performed an unbiased genome-wide survey to identify novel candidate genes that may be involved in statin response using genome-wide mRNA expression analysis in a sequential filtering strategy to identify those most likely to be relevant to cholesterol metabolism based on their gene expression characteristics. Among these, *RHOA* was selected for further functional study. A role for this gene in the maintenance of intracellular cholesterol homeostasis was confirmed by knock-down in hepatoma cell lines. In addition, statin-induced *RHOA* transcript levels measured in a panel of lymphoblastoid cell lines was correlated with statin-induced change in plasma LDL-cholesterol measured in individuals from whom the cell lines were derived. Lastly, a cis-acting splicing QTL associated with expression of a rare cryptic *RHOA* exon was also associated with statin-induced changes in plasma LDLC levels. This result exemplifies the power of applying biological information of well understood molecular pathways with genome-wide expression data for the identification of candidate genes that influence drug response.

## Introduction

Genome-wide association studies (GWAS) have been used to identify genetic contributors to a number of common diseases and traits [1]. However, a major problem with this approach is that very large sample sizes are generally required to detect statistically significant associations [2]. This is especially the case for pharmacogenomics, where identification of gene variants associated with drug response may require larger sample sizes than are generally available. Consequently, GWAS has had limited success in the identification of pharmacogenetically relevant single nucleotide polymorphisms (SNPs) that survive the stringency of genome-wide multiple testing [3], [4]. In the largest single statin clinical trial GWAS published to date (the JUPITER trial of ∼7000 individuals) only three loci (*ABCG2*, *APOE* and *LPA*) achieved genome-wide significance for association with the magnitude of LDL cholesterol reduction, and in total accounted for only a minor fraction of the overall variation in response [5]. Moreover, GWAS studies are limited by their ability to probe only common genetic variation and thus the limited findings suggest that association studies alone are unlikely to yield the basis for all or even the majority of the genetic variance associated with drug response.

In the present report, we describe the use of transcriptome-wide profiling to identify and prioritize genes that may contribute to inter-individual variation in statin-induced plasma LDL-cholesterol lowering. Statins inhibit HMG-CoA reductase (HMGCR), the enzyme that catalyzes the rate limiting step of cholesterol biosynthesis, thus lowering intracellular cholesterol levels [6]. This in turn elicits an increase in expression of cellular LDL receptors that mediate plasma LDL clearance [7]. Since the *HMGCR* gene is transcriptionally regulated by intracellular sterol content [8], the magnitude of induction of this gene is a cellular marker of *in vitro* statin response. We used expression array data from *in vitro* statin-exposed immortalized human hepatoma cell lines and lymphoblastoid cell lines established from participants of the Cholesterol and Pharmacogenetics (CAP) clinical trial of simvastatin treatment [9] to establish a set of “biological rules” for identifying genes whose expression characteristics qualified them as having biologically plausible effects on cholesterol metabolism. *RHOA* emerged from this analysis, and subsequent functional and genetic studies, as a novel candidate gene contributing to variation in LDL response to statins.

## Results

### Identification of *RHOA* as a candidate gene

**Figure 1:**
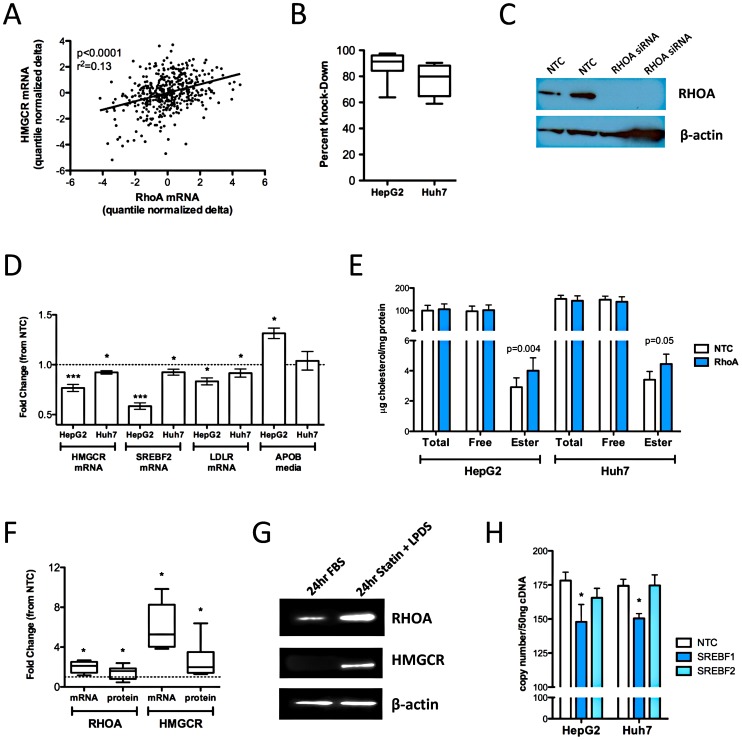
Functional validation of RHOA. (A) *HMGCR* and *RHOA* levels were quantified in 480 CAP LCLs after 24 hour incubation with 2 µM activated simvastatin or sham buffer. HepG2 (n = 12) and Huh7 cells (n = 6) cells were reverse transfected with Silencer Select siRNAs specific for *RHOA* or a negative control using the NeoSport transfection reagent. (B) After 48 hours, *RHOA* transcript levels were quantified by real time qPCR and compared to cells treated with the negative control to calculate percent knock-down of the *RHOA* transcript. (C) HepG2 cells (n = 2) were transfected and the cell lysate probed by immunoblot with antibodies to RHOA and β-actin. (D) *HMGCR*, *SREBF2*, and *LDLR* transcript levels were quantified by qPCR. APOB and APOAI accumulated in the cell culture media were quantified by ELISA. (E) Total and free intracellular cholesterol levels were quantified using the Amplex Red Cholesterol Assay Kit (Invitrogen), with cholesterol ester calculated as the difference of total minus free cholesterol. (F) HepG2 cells (n = 10) were incubated for 24 hours with 2 µM simvastatin +10% LPDS or sham buffer +10% FBS (control conditions), after which cells were harvested and *RHOA* and *HMGCR* mRNA were quantified by qPCR, and RHOA and HMGCR protein were quantified by immunoblot. Band densities were quantified using Alphaview SA version 3.4.0. The dashed line indicates a fold change of 1. (G) One representative immunoblot of HepG2 cells incubated as described in section D. (H) HepG2 and Huh7 cells (n = 6 each cell type) were transfected with the Silencer Select siRNA specific for *SREBF1, SREBF2* or a non-targeting negative control. *RHOA, HMGCR* and *CLPTM1* transcript levels were quantified after 48 hours. Statistically significant differences in gene expression, APOB, APOAI and intracellular cholesterol were evaluated by paired two-tailed t-test. All values shown are mean ± standard error. *p<0.05; **p<0.01, ***p<0.001.

We used a series of filters applied to genome-wide gene expression data from 480 human lymphoblastoid cell lines (LCLs) derived from participants in the Cholesterol and Pharmacogenetics study to identify genes that appeared to be biologically plausible candidates for modulating the effects of statins on cholesterol metabolism. The following filter criteria were used (Table 1): 1) expression in normal human liver; 2) change in transcript levels in HepG2 (n = 4) and Hep3B (n = 3) human hepatoma cell lines incubated with 2.0 µM activated simvastatin versus sham buffer for 24 hr, FDR<0.01, 3) change in transcript levels in CAP LCLs incubated with 2.0 µM activated simvastatin versus sham buffer for 24 hr (Q<0.05); 4) consistent directionality of statin-induced change in transcripts in hepatoma cell lines and LCLs; 5) correlation of statin-induced gene expression change in CAP LCLs with change in expression of *HMGCR*. After Bonferroni correction for multiple testing (p<1.17e-04) we identified 45 genes which passed all filter criteria (Table 2). When ranked in order of correlation, only two of the top thirteen genes did not encode enzymes in the cholesterol biosynthesis pathway: transmembrane protein 97 (*TMEM97*) and ras homolog gene family member A (*RHOA*). Although both had been previously implicated in lipid metabolism [10], [11], [12], neither had been shown to play a role in the cholesterol lowering effects of statin. However, *RHOA* was particularly intriguing since inhibition of RHOA signaling is thought to be a major mechanism by which statins exert pleiotropic (or non-lipid lowering) actions, such as anti-inflammatory effects. Figure 1A demonstrates the strong correlation between statin-induced change in *RHOA* and *HMGCR* transcript levels (p = 7.64E-16, r^2^ = 0.13).

**Table 1:** Identification of candidate genes associated with statin.

Expression Characteristics	Genes
Expressed in lymphoblastoid cell lines	10,149
Expressed in normal human liver, p<0.05	6629
Statin responsive in hepatoma cell lines, FDR<0.01	845
Statin responsive in LCLs, Q<0.05	725
Same direction of statin response	427
Statin-induced expression correlated with statin-induced change in *HMGCR*,	
Bonferroni (p<0.000117)	**45**

HepG2 (n = 4), Hep3B (n = 3) and 480 LCLs derived from participants of the Cholesterol and Pharmacogenetics (CAP) clinical trial were exposed to either 2 µM activated simvastatin or sham buffer for 24 hours, and gene expression was quantified on the Illumina HTref8v3 bead chip. Genes expressed in both LCLs and normal human liver (n = 60, GEO dataset GSE28893) were identified and tested for evidence of statin-induced changes in gene expression.

**Table 2:** Expression characteristics of identified candidate genes.

	HepG2 or 3B (n = 7)		CAP LCLs (n = 480)		Correlation with HMGCR	
Gene Name	Fold Change	FDR	Fold Change	Q-value	p-value	r
HMGCS1	1.75±0.13	0	1.56±0.42	0	1.76E-40	0.50
SQLE	1.59±0.09	0	1.37±0.29	0	1.51E-26	0.46
DHCR7	1.82±0.03	0	1.42±0.33	0	1.55E-26	0.36
SC4MOL	2.07±0.15	2.21E-03	1.53±0.70	0	2.27E-26	0.43
TM7SF2	1.65±0.18	5.54E-03	1.65±0.49	0	4.33E-26	0.40
LSS	1.88±0.07	0	1.51±0.37	0	1.59E-23	0.35
MVK	1.80±0.17	2.21E-03	1.32±0.30	0	1.83E-19	0.37
MVD	2.15±0.15	4.56E-03	1.38±0.35	0	1.35E-17	0.31
TMEM97	1.49±0.04	0	1.19±0.18	0	9.72E-17	0.29
NSDHL	1.53±0.07	0	1.19±0.25	0	5.68E-16	0.35
RHOA	1.47±0.09	2.67E-03	1.32±0.47	0	7.64E-16	0.39
EBP	1.42±0.09	8.81E-03	1.20±0.24	0	1.32E-14	0.30
FDFT1	1.48±0.07	0	1.19±0.19	0	1.42E-14	0.32
MSI2	0.83±0.02	0	0.96±0.14	0	3.68E-14	−0.23
FADS1	1.52±0.14	9.97E-03	1.24±0.31	0	2.96E-13	0.23

### Effects of RHOA on intracellular cholesterol homeostasis

To determine if RHOA has a direct effect on markers of intracellular cholesterol homeostasis, we transfected HepG2 cells (n = 10) with siRNAs specific for *RHOA* or a non-targeting negative control and tested for changes in expression of *HMGCR*, low-density lipoprotein receptor (*LDLR*) and sterol response element binding transcription factor (*SREBF2* aka *SREBP2*) gene expression. Knock-down reduced *RHOA* transcript levels by 60-98% (Figure 1B), with no remaining detectable RHOA protein (Figure 1C), and also generated statistically significant reductions in expression of *HMGCR* (0.76±0.04 fold, p = 0.002), *SREBF2* (0.58±0.03 fold, p = 0.0003), and *LDLR* (0.73±0.13 fold, p = 0.03) Figure 1D. *RHOA* knockdown-mediated reductions in expression of *HMGCR, LDLR*, and *SREBF2* were confirmed in a second hepatoma cell line, Huh7 (n = 6); however, the magnitude of the effect was less dramatic than that observed in the HepG2 transfections.

To further test the functional role of RHOA, we also measured levels of secreted APOB and APOA1, the major proteins on LDL and HDL particles respectively, in the culture media 48 hours after knock-down. APOB accumulation in the cell culture media was increased in HepG2 cells after *RHOA* knock-down (1.28±0.08 fold, p = 0.03, n = 12), while a similar but non-statistically significant trend was observed in Huh7 cells (1.08±0.06 fold, p = 0.10, n = 8), (Figure 1D). No significant changes in secreted levels of APOA1 were observed in either hepatoma cell line. Reduced *HMGCR*, *LDLR*, and *SREBF2* transcript levels together with increased APOB secretion with RHOA knock-down are all consistent with higher intracellular cholesterol levels, which was documented in the case of cholesterol esters (1.56±0.18 fold vs. controls, p = 0.004, n = 16), (Figure 1E). Although we also detected a trend for elevated free cholesterol after knock-down, this was not statistically significant. A trend of increased intracellular cholesterol ester and free cholesterol was also observed in Huh7 cells after *RHOA* knock-down (1.15±0.14 fold, p = 0.05 and 1.06±0.15 fold, p = 0.27, n = 8).

Lastly, since many genes involved in the maintenance of intracellular cholesterol are transcriptionally regulated in response to changes in intracellular sterol content through SREBF2, a transcription factor, we sought to test if *RHOA* was also subject to SREBF2 regulation. Sterol depletion activates SREBF2, thus stimulating expression of SREBF2 target genes. We confirmed that *RHOA* mRNA and protein levels were substantially increased by extreme sterol depletion in HepG2 cells with 2 µM simvastatin +10% lipoprotein deficient serum for 24 hr (Figure 1F and 1G). Induction of *HMGCR* mRNA and protein levels served as a positive control for the effects of cholesterol depletion. Finally, we found small but statistically significant reductions in *RHOA* transcript levels after *SREBF1* knock-down in HepG2 (0.83±0.07 fold, p = 0.05) and Huh7 (0.86±0.02 fold, p = 0.001) cell lines (Figure 1H).

### Correlation of *RHOA* transcript levels with cellular and clinical measures of statin response

**Figure 2:**
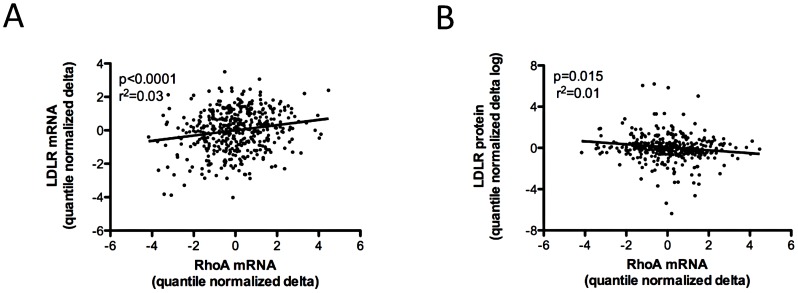
Association of variation in *RHOA* mRNA expression levels with other cellular phenotypes. 480 CAP LCLs from Caucasian donors were incubated with 2 µM activated simvastatin or sham buffer for 24 hours, after which genome-wide gene expression was quantified using the Illumina human HT8v3 beadarray, and cell surface LDLR protein was measured by FACS. Greater induction of *RHOA* transcript levels was positively correlated with greater induction of both *LDLR* and *HMGCR* (shown in Figure 1) mRNA (A) and inversely correlated with induction of cell surface LDLR protein (B). Gene expression values and measures of LDLR protein were adjusted for cellular covariates as described in Figure 1, log transformed, the delta calculated as the statin-treated minus the sham-treated value, and quantile normalized.

Although statin-induced changes of *RHOA* and *LDLR* mRNA were positively correlated in the LCL panel (Figure 2A), change of the *RHOA* transcript was inversely correlated with level of LDLR cell surface protein (Figure 2B). Consistent with this relationship, we also identified an inverse correlation of *RHOA* transcript levels in statin- treated CAP LCLs with absolute changes in plasma total cholesterol (p = 0.02, r^2^ = 0.01), LDL cholesterol (p = 0.04, r^2^ = 0.01) and APOB (p = 0.007, r^2^ = 0.01), measured *in vivo* before and after simvastatin treatment of the individuals from whom these cell lines were derived (Table S1). In contrast, levels of *RHOA* in sham-treated LCLs were not significantly correlated with these measures at baseline (Table S1). Moreover, *RHOA* transcript levels in statin-treated LCLs were not significantly associated with statin-induced changes in plasma HDL cholesterol levels (data not shown).

### A *RHOA* cis-splicing QTL is associated with *in vivo* statin response and exhibits allele-specific expression

**Figure 3:**
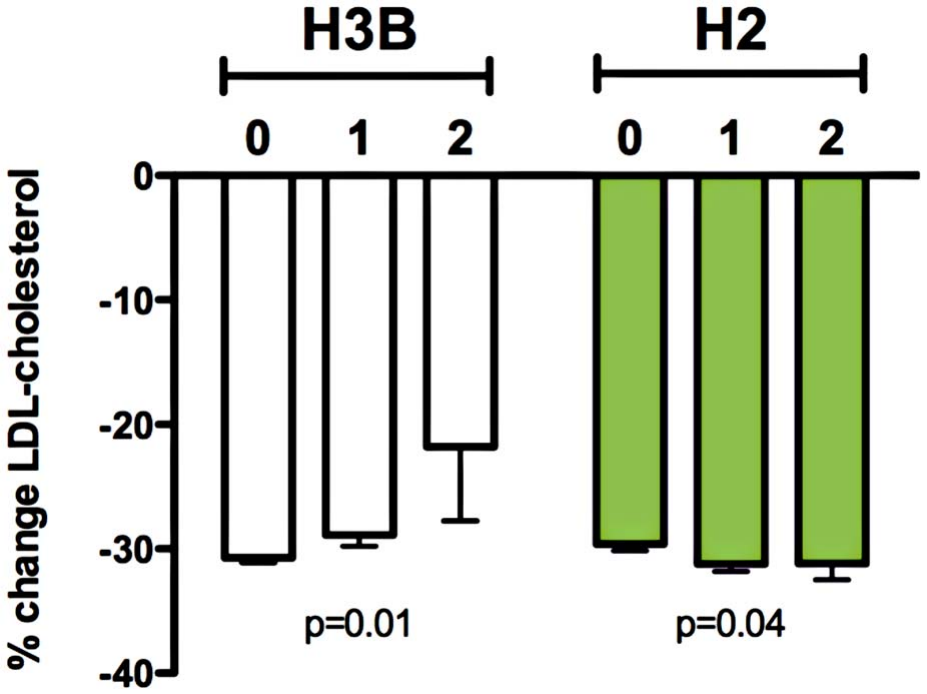
Relationship between *RHOA* haplotypes H3B and H2 and statin-induced changes in LDL-cholesterol response in the CAP (N = 580) and PRINCE (N = 1306) clinical trials. Linear regression of statin-induced changes (delta log) in LDL-cholesterol with adjustment age, sex, BMI, smoking status, and clinical trial was used to test for association with *RHOA* haplotypes. Graph depicts mean percent change in LDL-cholesterol +/− SE.

We next investigated the association of common genetic variation near *RHOA* with *in vivo* statin response. Analysis of HapMap3 CEU data [13] with Haploview [14], revealed that *RHOA* fell within a large block of linkage disequilibrium spanning almost 500 kb and that there were four major haplotypes when considering markers within 10 kb of the gene, all with frequencies greater than 10% in the CEU population (Figure S1; Table 3). Haplotypes were inferred based on directly genotyped SNPs (Table S2) or imputed genotypes (rs11716445 for H3B), and the number of copies of each haplotype were tested for association with change in LDL-cholesterol (delta log) in response to statin treatment of Caucasian participants in CAP (n = 580) and in the Pravastatin Inflammation CRP Evaluation (PRINCE: pravastatin 40 mg/day, 24 weeks, n = 1306) clinical trial, with adjustment for sex, age, BMI, smoking status, and study population. Of the four haplotypes, H3B showed the strongest association with statin response (p = 0.01), with homozygous H3B carriers having a 29% smaller reduction in the unadjusted percent change of LDL-cholesterol compared to non-carrier controls (−21.8±4.5% versus −30.7±0.4%, Figure 3). When the CAP and PRINCE cohorts were analyzed independently, the directionality of this association was consistent between the two populations (Figure S2). Haplotype H2 also demonstrated a modest association, with carriers having greater statin-induced changes in LDL-C (p = 0.04, n = 1886, Figure 3). There were no significant associations of H3B or H2 carrier status with baseline LDL-cholesterol (p = 0.3 for both).

**Table 3:** *RHOA* haplotype definition.

Haplotype	rs6446272	rs1464569	rs1464566	rs1464567	rs4855873	rs1464568	rs10640	rs9814873	rs6997	rs6784820	rs6779524	rs940045	rs13096474	rs2140270	rs6797765	rs4955412	rs7621003	rs17595772	rs11716445	rs974495	rs3448	rs3811699	rs17650792	rs17080528	N (CAP)	N (PRINCE)	%
**H1**	C	C	***G***	***C***	***G***	***T***	C	T	G	***C***	G	T	G	T	***C***	***G***	***G***	***T***	C	G	G	A	***C***	G	476	1154	42.6
**H2**	C	***T***	A	G	T	C	C	T	G	T	G	***C***	***A***	***C***	T	T	A	C	C	***A***	***A***	A	T	G	311	671	25.6
**H3A**	***T***	C	A	G	T	C	***T***	***C***	***A***	T	***A***	T	G	T	T	T	A	C	C	G	G	***G***	T	***A***	282	576	22.4
**H3B**	***T***	C	A	G	T	C	***T***	***C***	***A***	T	***A***	T	G	T	T	T	A	C	***T***	G	G	***G***	T	***A***	105	255	9.4

*RHOA* haplotypes were defined using common HapMap3 SNPs within 10 kb of the gene, with minor alleles in bold italics and the minor allele of rs11716445, the only *RHOA* SNP that differs between H3A and H3B, in underlined bold italic. Distributions of the haplotypes in the CAP and PRINCE clinical trial populations are also shown.

**Figure 4:**
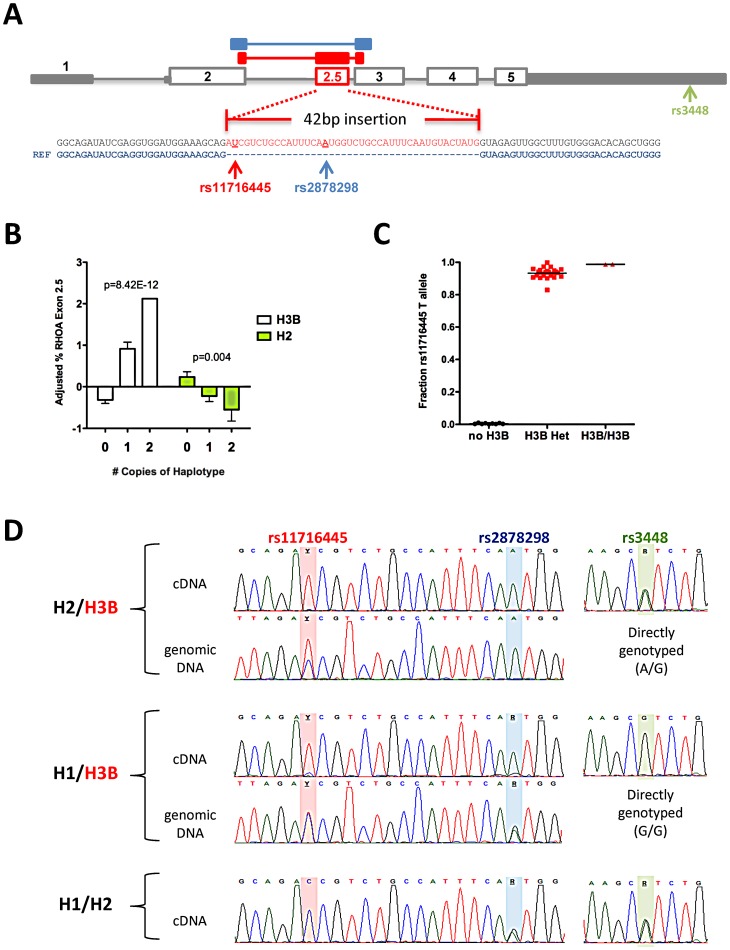
Association between *RHOA* haplotypes and expression of *RHOA* exon 2.5. (A) *RHOA* gene structure and location and sequence of exon 2.5. The 5′ and 3′ UTRs are indicated in solid grey, canonical exons are outlined in grey, and the novel exon is shown in red. The location of the three common *RHOA* exonic SNPs are shown. RNA-Seq libraries were prepared from HepG2, Hep3B, Huh7, and CAP LCLs (n = 3) and sequenced using 100 bp paired-end reads, and the novel exon was discovered by inspecting novel junction spanning reads. (B) Total *RHOA* expression levels and *RHOA* exon 2.5 levels were measured using qPCR, the percent containing exon 2.5 were calculated by dividing *RHOA* exon 2.5 levels by total *RHOA* levels, adjusted for batch effects using regression, and normal quantile transformed prior to association testing using linear regression. Graphs depict mean +/− SE. (C) CAP LCL cDNAs containing *RHOA* exon 2.5 were PCR-amplified and Sanger sequenced and the relative amounts of each allele of rs11716445 in exon 2.5 were estimated by measuring the height of the sequencing traces. (D) Representative sequencing traces of the LCL cDNA and genomic DNA of three heterozygous individuals from CAP illustrate that H3B is a cis-acting splicing QTL exhibiting allele-specific expression in *RHOA* exon 2.5 (rs11716445 and rs2878298) but not exon 5 (rs3448). H3B contains the minor allele [T] of rs11716445, H1 contains the minor allele [G] of rs2878298, and H2 contains the minor allele [A] of rs3448.

We found no association of either H3B or H2 with *RHOA* transcript levels in CAP LCLs after treatment with 2 µM statin or sham buffer (n = 115) (Figure S3A). However, rs11716445, the SNP that defines the H3B haplotype, is located in a rare 45 bp cryptic exon (referred to as *RHOA* exon 2.5) that we identified in multiple unique sequences during RNA-Seq analysis of three human hepatoma cell lines (HepG2, Hep3B and Huh7), and CAP LCLs (n = 3), Figure 4A. Expression of the *RHOA 2.5* exon was validated by Sanger sequencing. Notably, we found that the H3B haplotype showed a very strong association with *RHOA* exon 2.5 levels under both sham (p = 2.7×10^−7^, n = 119) and statin (p = 9.1×10^−13^, n = 115) conditions, with carriers exhibiting the highest levels of exon 2.5 inclusion (Figure 4B and Figure S3B). The H2 haplotype also exhibited a more modest association with *RHOA* exon 2.5 levels in the opposite direction from H3B (p<0.01), consistent with their *in vivo* relationships.

Using Sanger sequencing, we found evidence of allele-specific expression (ASE) at rs11716445 with over 90% of the exon 2.5-containing transcripts originating from the H3B chromosome (Figure 4C and 4D). We also observed evidence of ASE at rs2878298, another SNP found within exon 2.5 (Figure 4D and Figure S3C). There was no significant difference in the relative amount of ASE between the statin- and sham-treated states. Finally, to determine if rs11716445 was a general expression quantitative trait locus (eQTL) or a splicing QTL, we tested for ASE at rs3448, a SNP in the 3′ UTR of *RHOA*, in eight heterozygous carriers (or H2/H3B) and found no evidence that the ASE extended beyond exon 2.5 (Figure 4D and Figure S3C). These results strongly suggest that rs11716445 is a cis-acting splicing QTL.

## Discussion

We here present results of applying a set of biologically meaningful filters to identify and rank candidate genes associated with inter-individual variation in statin effects on cholesterol metabolism based on their gene expression characteristics. Using unbiased genome-wide screens, we identified genes that were normally expressed in the human liver and changed in response to statin treatment in a manner that was correlated with statin-induced change in *HMGCR* quantified as an *in vitro* marker of statin response. From these analyses we identified a number of genes not previously implicated in the lipid-lowering response to statin as potential candidates for future study. We selected *RHOA* since many of the non-lipid lowering benefits, or pleiotropic effects, of statin treatment have been attributed to its ability to inhibit RHOA activity. Our validation of RHOA as a modulator of cellular cholesterol metabolism, as well as the discovery that genetic variation within *RHOA* is associated with the magnitude of LDL-cholesterol response to statin treatment, support the continued studies of other novel candidate genes identified through this integrative genomics strategy.

RHOA has been previously implicated in cholesterol metabolism through the modulation of ABCA1-mediated cholesterol efflux via two distinct and opposing mechanisms. RHOA inhibition stimulates *ABCA1* gene expression via PPARγ and LXR activation [15], while RHOA activation increases ABCA1 protein stability [11]. Although excess intracellular levels of free cholesterol have been shown to increase RHOA activity [16], here we demonstrate that *RHOA* knock-down results in increased levels of secreted APOB, suggesting that RHOA may influence the pool of intracellular cholesterol available for lipoprotein production. Consistent with this hypothesis, we found that knock-down of *RHOA* in hepatoma cell lines resulted in increased intracellular content of cholesterol esters, the storage form of cellular cholesterol that can be mobilized for lipoprotein secretion. This occurred in conjunction with reduced expression of *HMGCR* and *LDLR*, presumably due to cholesterol-induced down-regulation of SREBF2. Very recently a novel protein, LAMTOR1 (also known as Pdro/p27RF-Rho), was found to both activate RHOA [17], and to regulate LDL-C uptake and intracellular cholesterol egress from the late endosome/lysosome [10], further supporting a link between RHOA and cholesterol metabolism.

Additional evidence for such a link is provided by the strong correlations that we observed between statin-induced changes in *RHOA* mRNA levels and both *HMGCR* and *LDLR* transcripts. On the other hand, there was an inverse correlation between change in *RHOA* mRNA and cell surface LDLR protein. While this may appear to be at odds with the change in *LDLR* transcript level, it is consistent with our finding that greater statin-induced *RHOA* gene expression was associated with reduced *in vivo* response lipid response to statin treatment. It is possible that increased RHOA expression directly or indirectly reduces functional LDLR at the cell surface by altering post-translational processing or cellular trafficking, hypotheses that will be tested in future studies. Increased magnitude of this effect may contribute to attenuation of statin-induced plasma LDL lowering.

Based on its role in mediating the pleiotropic effects of statin response, *RHOA* has been proposed as a candidate gene for the study of statin pharmacogenetics; however, genetic variation within *RHOA* associated with statin response has not been previously identified [18]. Here, we report that a common *RHOA* haplotype, H3B, is associated with reduced LDL-cholesterol lowering in response to statin treatment in data derived from two independent clinical trials. Within *RHOA*, this haplotype was defined by a single SNP, rs11716445; however, since rs11716445 is in strong linkage disequilibrium with many SNPs in other genes up to 500 kb away from *RHOA*, it is possible that its association with statin response may also be due to genetic variation affecting other genes. rs11716445 explained less than 1% of the overall variation in LDL cholesterol response to statin, so neither the H3B haplotype or rs11716445 genotype alone would be a clinically useful diagnostic, but it could be included with other known markers of statin response to improve prediction algorithms.

Here we demonstrate that rs11716445 is a cis-acting splicing QTL also associated with allele-specific expression of *RHOA* exon 2.5, a rare exon found within *RHOA* intron 2. The presence of this exon does not disrupt the open reading frame and is predicted to cause a 14 amino acid inclusion in the B3 domain of the RHOA protein, a region with no known interactions [19], [20]. Although the functional impact of exon 2.5 inclusion is unknown, the fact that the two *RHOA* haplotypes associated with its expression levels, H3B and H2, are also the only two *RHOA* haplotypes found to be associated with *in vivo* variation in statin-induced change in LDL-cholesterol, strongly supports the likelihood that *RHOA* alternative splicing is functionally relevant.

*In silico* analysis with ESEfinder 3.0 identified SRSF2 (aka SC35), SRSF5 (aka SRp40), and SRSF1 (aka SF2/ASF) binding sites within 20 bp of the exon 2.5 splice donor [21]. Notably, the rs11716445 “T” allele is predicted to disrupt an SRSF5 binding motif (TAGA[T/C]C) (Figure S4). This finding is consistent with previous reports demonstrating that SRSF5 and SRSF2 antagonize SRSF1 to promote exon exclusion, as the loss of the SRSF5 binding with the “T” allele would be predicted to result in exon 2.5 inclusion [22]. Thus, these results strongly suggest that the rs11716445 “T” (minor) allele enhances expression of the *RHOA* 2.5 exon. We also found that the proportion of the expressed *RHOA* 2.5 exon containing the “T” allele was reduced in H1/H3B compared to H2/H3B and H3A/H3B heterozygotes (Figure S3C). Since the H1 haplotype contains the minor allele of the second common SNP within the 2.5 exon, rs2878298, which is predicted to generate a SRSF1 binding site, these findings suggest that there are multiple gene variants that regulate expression of this novel exon; however the functional effects of these SNPs (rs11716445 and rs2878298) as well as the expression of the cryptic *RHOA* exon remain to be tested.

In summary, we here report using a combination of expression array data, functional studies, and genetic analyses that *RHOA* is a novel candidate gene associated with variation in both *in vitro* and *in vivo* response to statin. Although additional studies of statin effects will be required to corroborate these findings, they demonstrate the value of using data from a variety of molecular techniques, including the combination of *in vivo* and *in vitro* genetically-regulated phenotypes, as a novel approach for identifying genes involved in drug response.

## Materials and Methods

### Cell culture

Lymphoblastoid cell lines (LCLs) from 480 Caucasian participants from the Cholesterol and Pharmacogeneomics (CAP) clinical trial [9] and HepG2 and Hep3B cell lines were grown under standard conditions and exposed to 2 µM simvastatin or sham buffer for 24 hours as previously described [23]. Although much higher than normal circulating levels of plasma simvastatin, 2–40 nM [24], this concentration of simvastatin was selected based on previous dose response experiments that were used to determine the amount that elicits a consistent and significant induction of both *HMGCR* and *LDLR* mRNA (Figure S5) [23]. Simvastatin was provided by Merck Inc. (Whitehouse Station, NJ) and activated to the β-hydroxyacid prior to use [25]. Cell surface LDLR protein was measured in statin and sham treated CAP LCLs as previously described [26]. To confirm statin regulation, HepG2 cells were grown in 6-well plates and incubated with 2 µM activated simvastatin +10% lipoprotein deficient serum (Hyclone) for 24 hours.

### Transcript quantification

Genome-wide gene expression was measured in RNA from CAP samples and statin and sham treated HepG2 and Hep3B cells. RNA was converted to biotin-labeled cRNA using the Illumina TotalPrep-96 RNA amplification kit (Applied Biosystems, Foster City, CA). cRNA was hybridized to Illumina HumanRef8v3 expression beadchips (Illumina, San Diego, CA). Data were analyzed using GenomeStudio (Illumina). All beadchips had a signal P95/P05>10. Significance analysis of microarrays (SAM) [27] was performed on the 10,291 of 18,630 probed genes that were expressed in LCLs (FDR<0.05). Expression traits were adjusted for known covariates (age, gender, exposure batch, cell growth rate as determined by cell count on exposure day, and RNA labeling batch) and also for unknown sources of variation through adjustment for those principal components that described greater than 5% variance across the dataset [28]. Adjusted data were quantile normalized across each gene to ensure normality.

Gene expression in human liver was determined using mean detection p-value as determined by GenomeStudio (Illumina, San Diego, CA) from expression profiles measured by Illumina Ref8v2 beadarray on 120 human liver samples (2 technical replicates each of 60 samples, GEO accession number: GSE28893 [29]). Mean detection p-values across all 120 samples was assessed, and genes with a p<0.05 were called expressed.

*HMGCR, LDLR*, *SREBF1, SREBF2, RHOA* (total), and *RHOAexon2.5* transcript levels were quantified by qPCR with gene expression normalized to *CLPTM* (TaqMan Assay number: Hs00171300_m1, Life Technologies) as previously described [30]. Primers used for qPCR of total *RHOA* were F: CGGAATGATGAGCACACAAG and R: TGCCTTCTTCAGGTTTCACC and those used for qPCR of *RHOA* exon 2.5 were F: TATCGAGGTGGATGGAAAGC and R: GCCAACTCTACCATAGTACATTGAAA.

### siRNA transfections

*RHOA*, *SREBF1* and *SREBF2* knock-down was achieved by 48 hour transfection of 80,000 HepG2 or Huh7 cells/well in 12-well plates using either the Ambion Silence Select siRNA (s759) or non-targeting control according to the manufacturer's protocol. Cell culture media was collected from all samples at time of harvest, and APOB and APOAI were quantified in triplicate by sandwich-style ELISA. Samples with a coefficient of variation greater than 15% were subject to repeat measurement. Cholesterol was extracted from the cell pellets with hexane-isopropanol (3∶2, v/v) and dried under nitrogen. The extracted cholesterol was reconstituted with reaction buffer (0.5 M potassium phosphate, pH 7.4, 0.25 M NaCl, 25 mM cholic acid, 0.5% Triton X-100). Total cholesterol content was determined with the Amplex Red Cholesterol Assay Kit (Invitrogen) and normalized to total cellular protein quantified by the Pierce BCA Protein Assay Kit (Thermo Scientific). To quantify RHOA protein levels, cells were lysed in Cell Lytic lysis buffer (Sigma), loaded on a 4–12% Tris-Glycine Gel (Invitrogen), and proteins were transferred onto a PDVF membrane using the iBLOT gel transfer system (Invitrogen). The blot was then probed with antibodies diluted 1∶200 to RHOA (SC26C4), HMGCR (SCH300) and β-actin (SC ACTBD11B7), all purchased from Santa Cruz Biotechnology. Band densities were analyzed using the Mulitplex Band Analysis tool in Alphaview SA version 3.4.0.

### Imputation and haplotype reconstruction

Haplotypes H1, H2, and H3A were assigned using genotype data from tag SNPs (Table S2), while haplotype H3B was inferred using imputed rs11716445 genotypes. Imputation was performed in BIMBAM using 317K or 610K genotypes in a similar manner as previously described [31] except for use of the HapMap3 and 1KGP CEU pilot data as a reference population.

### *In vivo* association analysis

LDL-cholesterol was quantified in self-reported Caucasian American participants of the Cholesterol and Pharmacogenetics (CAP) clinical trial twice at baseline and after both 4 weeks and 6 weeks of simvastatin 40 mg/day and in the participants of the Pravastatin Inflammation and CRP Evaluation (PRINCE) clinical trial after 12 and 24 weeks of pravastatin 20 mg/day as previously described [9], [32]. Delta log LDL-cholesterol was calculated as the log average value of LDL-cholesterol on treatment minus the log average of the two baseline measurements, and percent change was the average on-statin value minus the average baseline value over the average baseline value. The CAP trial is registered at ClinicalTrials.gov (NCT00451828). Informed consent was obtained and approved by the institutional review boards of the sites of recruitment, University of California Los Angeles and San Francisco General Hospital. In addition, all research involving human participants was approved by the Children's Hospital Oakland Research Institute IRB. All haplotypes with a minor allele frequency greater than 5% were identified using Haploview [14] with HapMap3 CEU data. Using an additive genetic model, haplotypes were tested for association with change in delta log LDL-cholesterol using combined results of both clinical trials with adjustment for age, sex, BMI, smoking status, and study population as well as for each trial separately with adjustment for age, sex, BMI, and smoking status.

### RNA–seq analysis

Hep3B, HepG2, and Huh7 cells were incubated in duplicate under either standard growth conditions (MEM supplemented with 10% FBS, 1% nonessential amino acids and 1% sodium pyruvate) or sterol depleted conditions (MEM supplemented with 1% nonessential amino acids, 1% sodium pyruvate, 2.0 µM simvastatin and 10% lipoprotein deficient serum) for 24 hours. RNA was extracted as previously described and samples from the duplicate experiments were pooled. Sequencing libraries were prepared by isolating mRNA from 7–10 µg total RNA using two rounds of the MicroPoly(A)Purist kit (Ambion), fragmenting the mRNA for 20 seconds, synthesizing cDNA using random primers, repairing ends, dA-tailing, ligating adapters, gel purifying fragments, amplifying libraries using indexed primers for 15 PCR cycles, and performing another round of gel purification. Libraries were sequenced to an average depth of 60 million 100 bp reads (30 million paired-end fragments). Expression of the novel *RHOA* exon was verified in independent samples through RT-PCR and Sanger sequencing.

### Sanger sequencing

DNA and RNA was isolated from CAP LCLs after 24 hours of exposure to sham buffer or 2 µM simvastatin. The DNA sequences of exon 2.5 and exon 5 were amplified using F: CAAGGCAGGAGAATGGTGTG and R: CCACTGACGATGATTGCTTC and F: GGCCATATTACCCCTTTTCG and R: CCAGAGGGATCTAGGCTTCC, respectively. RT-PCR was performed to amplify the transcript sequences of exon 2.5 and exon 5 (3′UTR) using F: TCGTTAGTCCACGGTCTGGT and R: GCCAACTCTACCATAGTACATTGAAA and F: CGGAATGATGAGCACACAAG and R: TTGGAAAAATTAACTGGTACAGAAA, respectively. PCR products were then subject to Sanger sequencing.

## Supporting Information

Figure S1 Linkage disequilibrium plot of the HapMap3 CEU population showing rs11716445 is in strong in LD with many other SNPs across a large region. Image was generated using SNAP [33].
(TIF)

Figure S2 Relationship between common *RHOA* haplotypes and statin-induced changes in LDL-cholesterol in the CAP and PRINCE clinical trials. *RHOA* haplotypes (A) H3B, (B) H2, (C) H1, and (D) H3A were tested for association with statin-induced changes in plasma LDL-cholesterol levels during the CAP and PRINCE statin clinical trials. P-values shown are for linear regression of delta log LDL-cholesterol adjusted for age, sex, BMI, smoking status, and study (for CAP+PRINCE analyses) versus number of copies of the haplotype. Graph depicts mean +/− SE.
(TIF)

Figure S3 Relationship between common *RHOA* haplotypes and *RHOA* transcript levels and splicing in CAP LCLs after treatment with 24 hr 2 uM simvastatin or sham buffer. (A) Total *RHOA* expression levels and (B) *RHOA* exon 2.5 levels were measured using qPCR, normalized against *CLPTM*, adjusted for batch effects using regression, and normal quantile transformed prior to testing for association with H3B or H2 copy number using linear regression. (C) CAP LCL cDNA containing *RHOA* exon 2.5 or a portion of exon 5 were PCR-amplified and Sanger sequenced and the relative amounts of each allele of the three common exonic *RHOA* SNPs were estimated by measuring the height of the sequencing traces in individuals with various combinations of haplotypes. H3B contains the minor allele [T] of rs11716445, H1 contains the minor allele [G] of rs2878298, and H2 contains the minor allele [A] of rs3448. Graphs depict mean +/− SE.
(TIF)

Figure S4 Predicted SRSF5 binding motif in *RHOA* exon 2.5. ESEfinder 3.0 [34]was used to identify putative splicing factor binding motifs disrupted by rs11716445.
(TIF)

Figure S5 Dose response curve of *HMGCR* and *LDLR* gene expression after incubation with activated simvastatin or sham buffer. (A) Data from eight CAP LCLs after 24 hr incubation. *HMGCR* and *LDLR* gene expression were quantified on the Illumina human HT8v3 beadarray. *Significantly different from sham treatment, p<0.05. (B) Data from HepG2 cell after 24 hour incubation, n = 6. *HMGCR* and *LDLR* gene expression were quantified by qPCR. Details regarding transcript quantification of both experiments are described in the Materials and Methods.
(TIF)

Table S1 P-values for association between *RHOA* transcript levels and plasma lipids. Total cholesterol, LDL cholesterol and APOB were quantified in 480 Caucasian American participants of the Cholesterol and Pharmacogenetics clinical trial twice at baseline and after both 4 weeks and 6 weeks of simvastatin 40 mg/day. Baseline values were averaged and on-treatment values were averaged, and the change (delta) was calculated as the percent difference. *RHOA* transcript levels were quantified in LCLs after 24 hr statin or sham treatment as described in Figure 1, and tested for correlation with plasma lipids using a multivariate fit model with adjustment for age, sex, smoking status and BMI. Shown are p-values for three types of associations: Baseline represents the association between baseline plasma lipids and *RHOA* transcript in the sham treated cells, Statin Treated represents the association between plasma lipids after statin treatment and *RHOA* transcript quantified in the statin treated cells, and Delta represents the association between statin-induced percent change in plasma lipids and statin-induced fold change of *RHOA* transcript levels. r^2^ values are shown for relationships with a p<0.05.
(DOCX)

Table S2 TagSNPs genotyped on the Illumina HumanHap 300 K and 610K-Quad platforms used to infer *RHOA* haplotypes H1, H2, and H3A, based on a proxy search using SNAP [33] in the HapMap3 CEU population. Coordinates are hg18.
(DOCX)
