# Correction: RHOA Is a Modulator of the Cholesterol-Lowering Effects of Statin

**DOI:** 10.1371/annotation/1c64490e-a511-4843-8061-8e7312c69568

**Published:** 2013-02-07

**Authors:** Marisa W. Medina, Elizabeth Theusch, Devesh Naidoo, Frederick Bauzon, Kristen Stevens, Lara M. Mangravite, Yu-Lin Kuang, Ronald M. Krauss

In the Funding section, one of the NIH grant numbers was listed incorrectly. The correct funding information is as follows: This work was supported by NHLBI R01 HL104133 and NHLBI U19 HL069757. The funders had no role in study design, data collection and analysis, decision to publish, or preparation of the manuscript.

