# Supplementary figures and images for: RHOA Is a Modulator of the Cholesterol-Lowering Effects of Statin
Source: PLoS Genet. 2012 Nov 15;8(11):e1003058. doi: 10.1371/journal.pgen.1003058 (PMC3499361; doi:10.1371/journal.pgen.1003058)

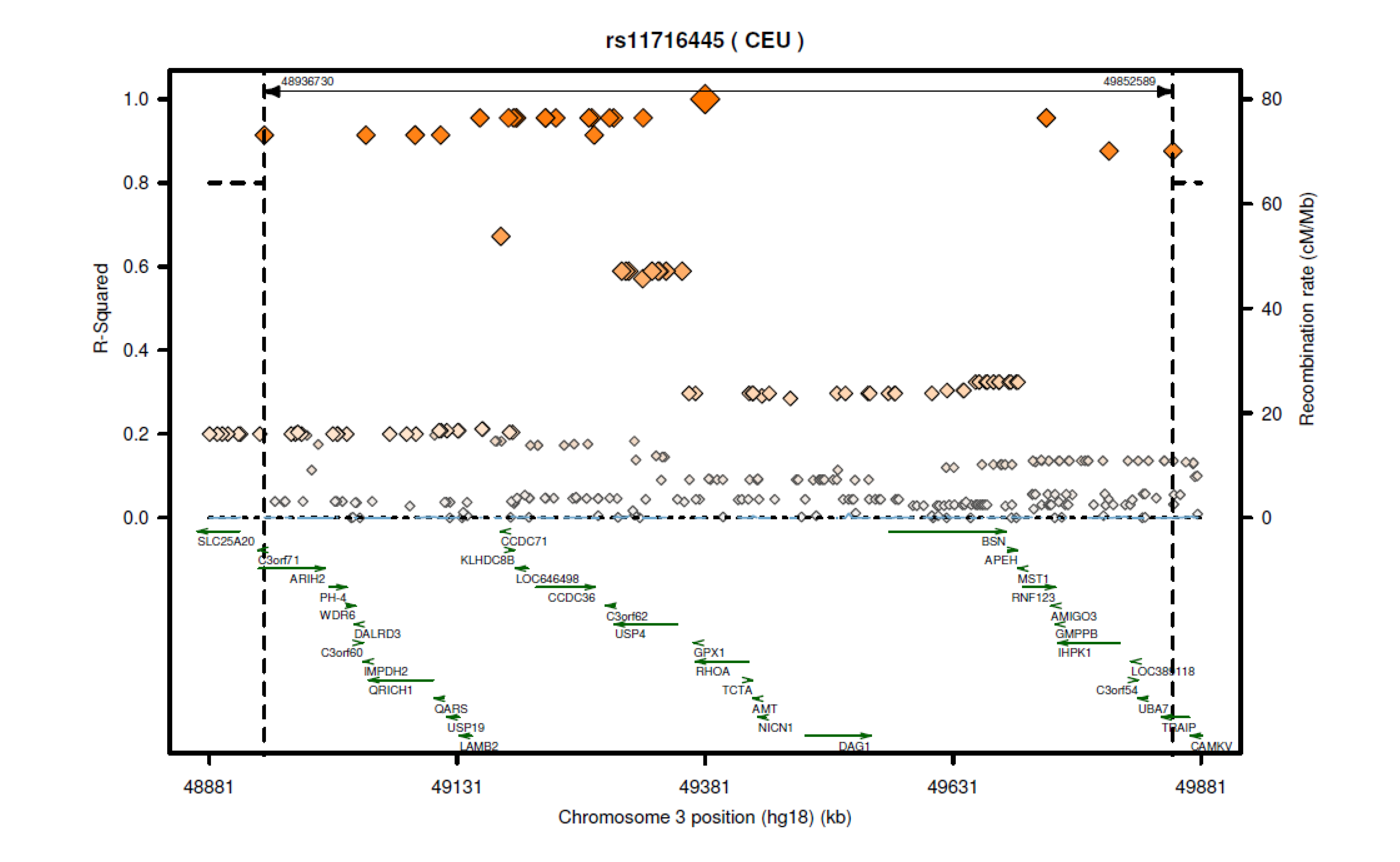

Supplement: Figure S1 — Linkage disequilibrium plot of the HapMap3 CEU population showing rs11716445 is in strong in LD with many other SNPs across a large region. Image was generated using SNAP [33]. (TIF) [file pgen.1003058.s001.tif]

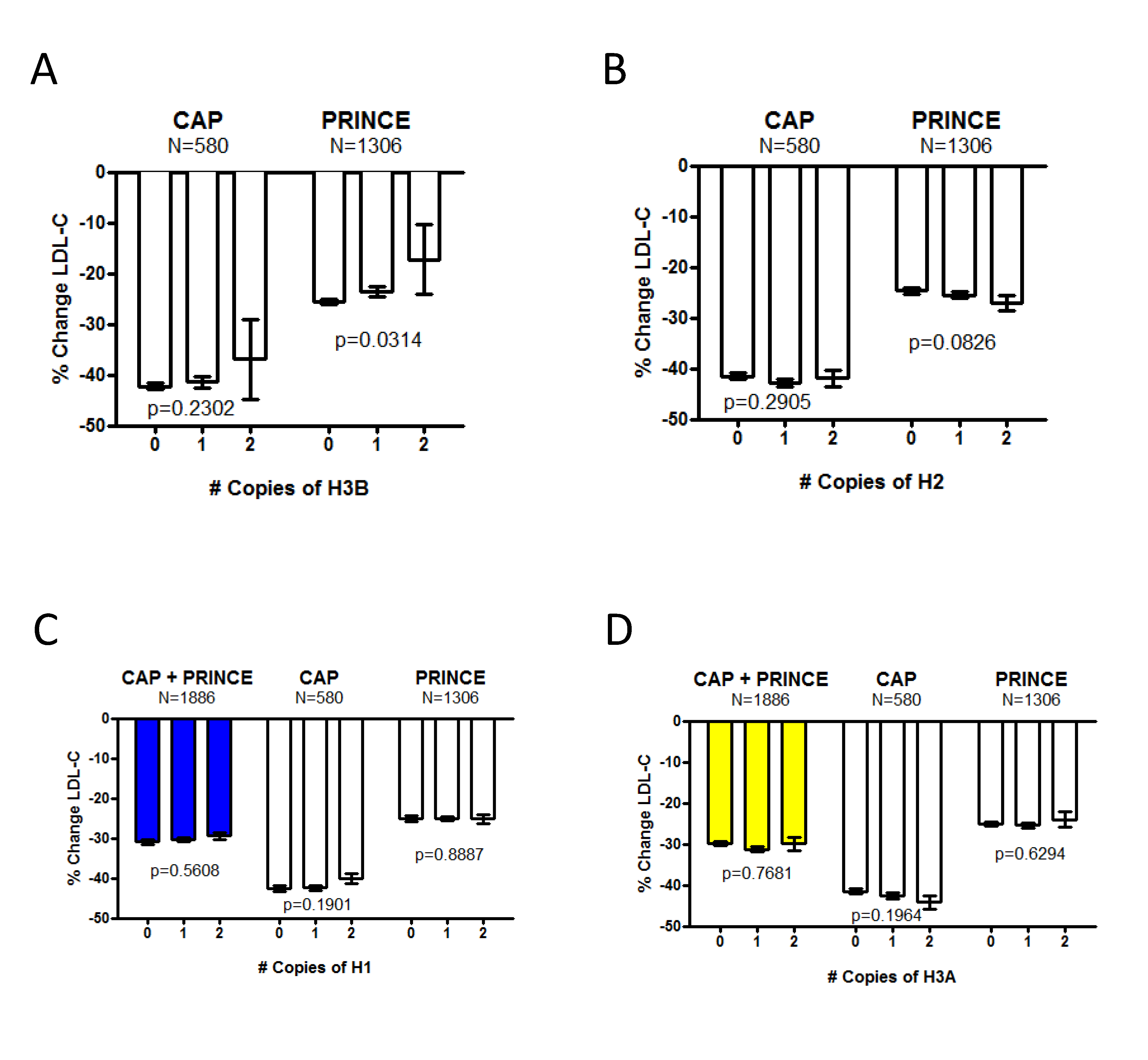

Supplement: Figure S2 — Relationship between common RHOA haplotypes and statin-induced changes in LDL-cholesterol in the CAP and PRINCE clinical trials. RHOA haplotypes (A) H3B, (B) H2, (C) H1, and (D) H3A were tested for association with statin-induced changes in plasma LDL-cholesterol levels during the CAP and PRINCE statin clinical trials. P-values shown are for linear regression of delta log LDL-cholesterol adjusted for age, sex, BMI, smoking status, and study (for CAP+PRINCE analyses) versus number of copies of the haplotype. Graph depicts mean +/− SE. (TIF) [file pgen.1003058.s002.tif]

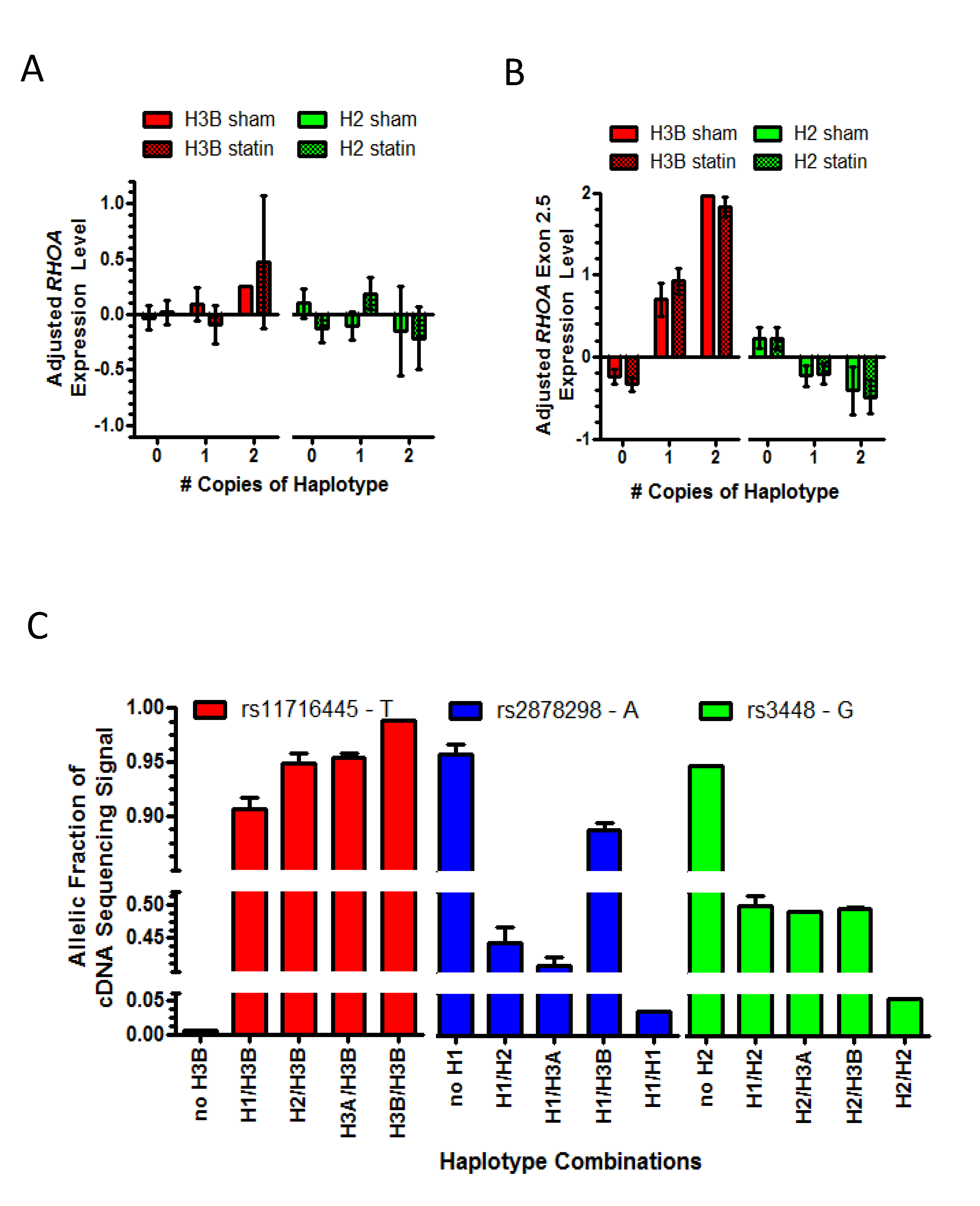

Supplement: Figure S3 — Relationship between common RHOA haplotypes and RHOA transcript levels and splicing in CAP LCLs after treatment with 24 hr 2 uM simvastatin or sham buffer. (A) Total RHOA expression levels and (B) RHOA exon 2.5 levels were measured using qPCR, normalized against CLPTM, adjusted for batch effects using regression, and normal quantile transformed prior to testing for association with H3B or H2 copy number using linear regression. (C) CAP LCL cDNA containing RHOA exon 2.5 or a portion of exon 5 were PCR-amplified and Sanger sequenced and the relative amounts of each allele of the three common exonic RHOA SNPs were estimated by measuring the height of the sequencing traces in individuals with various combinations of haplotypes. H3B contains the minor allele [T] of rs11716445, H1 contains the minor allele [G] of rs2878298, and H2 contains the minor allele [A] of rs3448. Graphs depict mean +/− SE. (TIF) [file pgen.1003058.s003.tif]

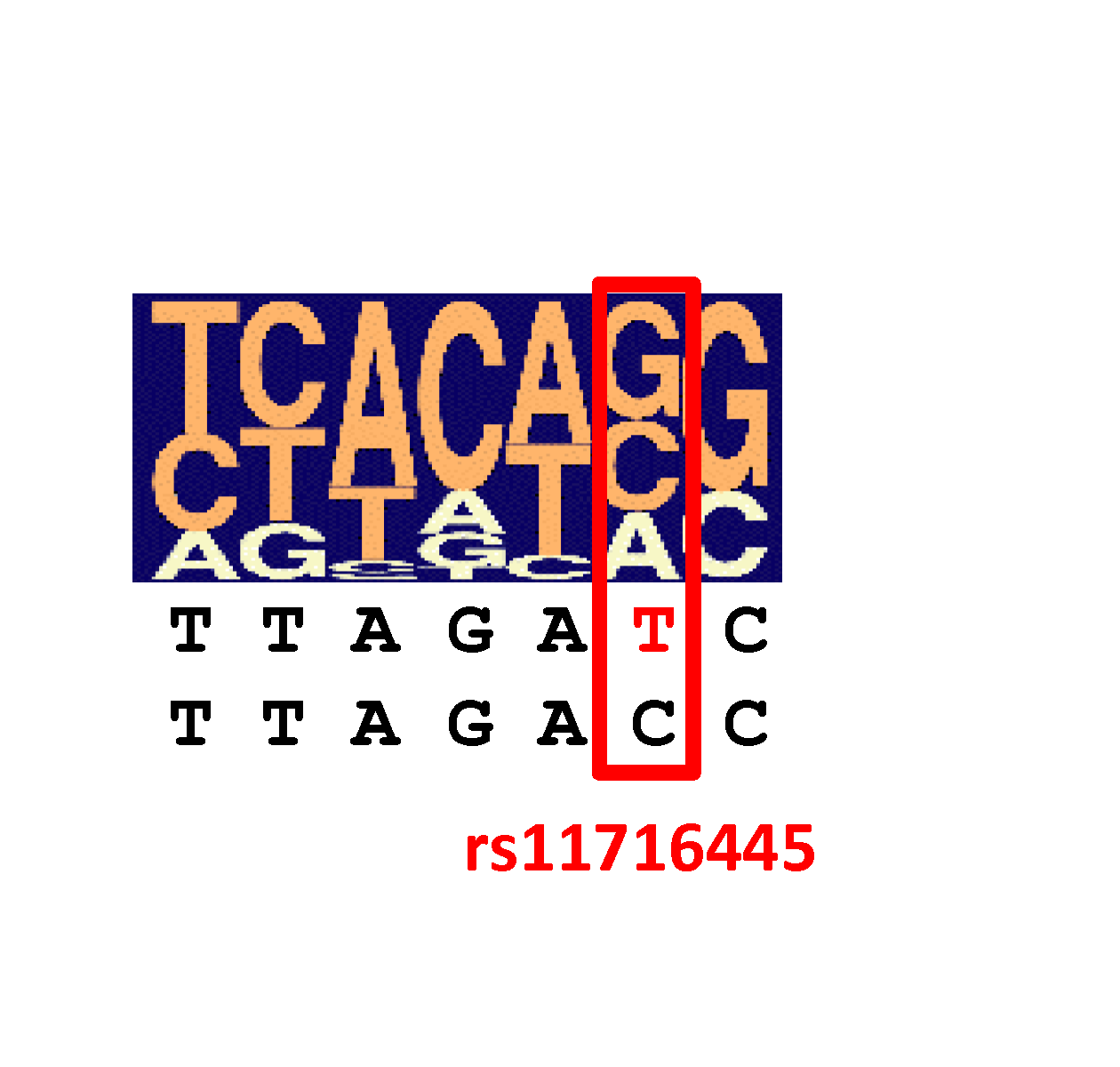

Supplement: Figure S4 — Predicted SRSF5 binding motif in RHOA exon 2.5. ESEfinder 3.0 [34]was used to identify putative splicing factor binding motifs disrupted by rs11716445. (TIF) [file pgen.1003058.s004.tif]

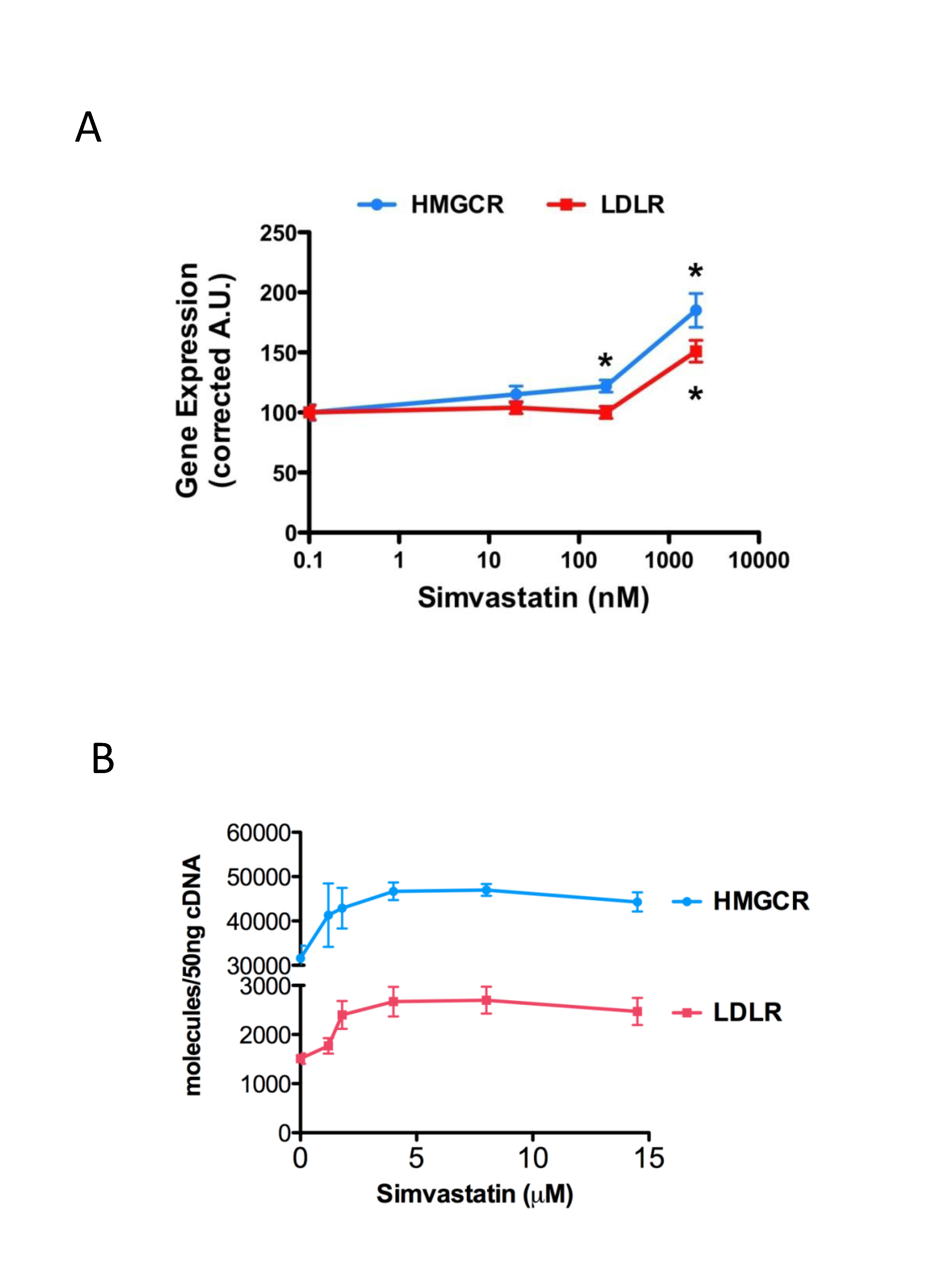

Supplement: Figure S5 — Dose response curve of HMGCR and LDLR gene expression after incubation with activated simvastatin or sham buffer. (A) Data from eight CAP LCLs after 24 hr incubation. HMGCR and LDLR gene expression were quantified on the Illumina human HT8v3 beadarray. *Significantly different from sham treatment, p<0.05. (B) Data from HepG2 cell after 24 hour incubation, n = 6. HMGCR and LDLR gene expression were quantified by qPCR. Details regarding transcript quantification of both experiments are described in the Materials and Methods. (TIF) [file pgen.1003058.s005.tif]
