# Supplementary material for: RHOA Is a Modulator of the Cholesterol-Lowering Effects of Statin
Source: PLoS Genet. 2012 Nov 15;8(11):e1003058. doi: 10.1371/journal.pgen.1003058 (PMC3499361; doi:10.1371/journal.pgen.1003058)
Supplement: Table S1 — P-values for association between RHOA transcript levels and plasma lipids. Total cholesterol, LDL cholesterol and APOB were quantified in 480 Caucasian American participants of the Cholesterol and Pharmacogenetics clinical trial twice at baseline and after both 4 weeks and 6 weeks of simvastatin 40 mg/day. Baseline values were averaged and on-treatment values were averaged, and the change (delta) was calculated as the percent difference. RHOA transcript levels were quantified in LCLs after 24 hr statin or sham treatment as described in Figure 1, and tested for correlation with plasma lipids using a multivariate fit model with adjustment for age, sex, smoking status and BMI. Shown are p-values for three types of associations: Baseline represents the association between baseline plasma lipids and RHOA transcript in the sham treated cells, Statin Treated represents the association between plasma lipids after statin treatment and RHOA transcript quantified in the statin treated cells, and Delta represents the association between statin-induced percent change in plasma lipids and statin-induced fold change of RHOA transcript levels. r2 values are shown for relationships with a p<0.05. (DOCX) [file pgen.1003058.s006.docx]

**Table S1**

|  | **Total Cholesterol** | | **LDL-Cholesterol** | | **ApoB** | |
| --- | --- | --- | --- | --- | --- | --- |
| **RHOA mRNA** | p-value | r^2^ | p-value | r^2^ | p-value | r^2^ |
| **Sham** | 0.18 |  | 0.15 |  | 0.13 |  |
| **Statin** | 0.02 | 0.01 | 0.04 | 0.01 | 0.007 | 0.01 |
| **Delta** | 0.25 |  | 0.17 |  | 0.16 |  |
