# Supplementary material for: RHOA Is a Modulator of the Cholesterol-Lowering Effects of Statin
Source: PLoS Genet. 2012 Nov 15;8(11):e1003058. doi: 10.1371/journal.pgen.1003058 (PMC3499361; doi:10.1371/journal.pgen.1003058)
Supplement: Table S2 — TagSNPs genotyped on the Illumina HumanHap 300 K and 610K-Quad platforms used to infer RHOA haplotypes H1, H2, and H3A, based on a proxy search using SNAP [33] in the HapMap3 CEU population. Coordinates are hg18. (DOCX) [file pgen.1003058.s007.docx]

**Table S2**

| **TagSNP** | **Haplotype** | **Chr** | **bp** | **r2** | **Major** | **Minor** | **MAF** |
| --- | --- | --- | --- | --- | --- | --- | --- |
| rs3448 | H2 | 3 | 49371755 | 1 | C | T | 28.0% |
| rs3924462 | H1 | 3 | 49499240 | 1 | T | G | 44.4% |
| rs11130199 | H1 | 3 | 49513803 | 1 | T | C | 44.4% |
| rs4625 | H3A | 3 | 49547144 | 1 | A | G | 27.6% |
| rs3866330 | H2 | 3 | 49557998 | 1 | C | T | 28.0% |
| rs4241407 | H2 | 3 | 49575323 | 1 | A | G | 28.0% |
